# Pulmonary recruitment maneuver reduces the intensity of post-laparoscopic shoulder pain: a systematic review and meta-analysis

**DOI:** 10.1186/s12871-023-02107-y

**Published:** 2023-05-04

**Authors:** Xiao Deng, Hao Li, Yantong Wan, Xuemei Lin

**Affiliations:** 1 Department of Anesthesiology, West China Second University Hospital, Sichuan University, Chengdu, Sichuan China; 2 Key Laboratory of Birth Defects and Related Diseases of Women and Children, Sichuan University, Ministry of Education, Chengdu, Sichuan China

**Keywords:** Pulmonary recruitment maneuver, PRM, Shoulder pain, Laparoscopy, Pain management

## Abstract

**Background:**

Post-laparoscopic shoulder pain (PLSP) is a common complication following laparoscopic surgeries. This meta-analysis aimed to investigate whether pulmonary recruitment maneuver (PRM) was beneficial to alleviated shoulder pain after laparoscopic procedures.

**Methods:**

We reviewed existing literature in the electronic database from the date of inception to January 31, 2022. The relevant RCTs were independently selected by two authors, after which data extraction, assessment of the risk of bias, and comparison of results.

**Results:**

This meta-analysis included 14 studies involving 1504 patients, among which 607 patients were offered pulmonary recruitment maneuver (PRM) alone or in combination with intraperitoneal saline instillation (IPSI), while 573 patients were treated with passive abdominal compression. The administration of PRM significantly decreased the post-laparoscopic shoulder pain score at 12 h (MD (95%CI) − 1.12(–1.57, − 0.66), n = 801, *P* < 0.001, I^2^ = 88%); 24 h (MD (95%CI) − 1.45(–1.74, − 1.16), n = 1180, *P* < 0.001, I^2^ = 78%) and at 48 h (MD (95%CI) − 0.97(–1.57, − 0.36), n = 780, *P* < 0.001, I^2^ = 85%). We observed high heterogeneity in the study and analyzed the sensitivity but failed to identify the cause of the heterogeneity, which may have resulted from the different methodologies and clinical factors in the included studies.

**Conclusion:**

This systematic review and meta-analysis indicate that PRM can reduce the intensity of PLSP. More studies may be needed to explore the usefulness of PRM in more laparoscopic operations besides gynecological surgeries and determine the optimal pressure of PRM or its appropriate combination with other measures. The results of this meta-analysis should be interpreted with caution owing to the high heterogeneity between the analyzed studies.

## Introduction

Laparoscopy is among the most used minimally invasive procedures that can reduce postoperative pain, lessen the duration of hospital stay and facilitate recovery earlier than laparotomy.Laparoscopy has been widely used in various abdominal surgeries, such as gastrectomy, cholecystectomy, appendectomy, hernia and gynecological surgery [1–5]. However, the post-laparoscopic shoulder pain (PLSP) is often occurs following laparoscopic surgeries, and its reported incidence varies from 35–80% [6–7]. The PLSP can even remain for up to three days and often upsets the patients [8]. Moreover, it can increase the costs of healthcare owing to an increased usage of analgesics, delayed discharge, and even re-admission [9]. Therefore, necessary measures should be taken to diminish the intensity of PLSP.

Although the exact mechanism of PLSP remains unclear, some studies have suggested that it is caused by the trapping of carbon dioxide (CO_2_) between the liver and the right diaphragm and subsequent conversion into carbonic acid, which irritates the diaphragm and subsequently generates referred shoulder pain (C4 dermatomal) [10–12]. Therefore, several studies have attempted to decrease the incidence or severity of PLSP by promoting the removal of remaining CO_2_ from the abdominal cavity. These efforts include drainage tube insertion, intraperitoneal saline instillation (IPSI), and the usage of intraperitoneal local anesthetic agents [13–15]. Moreover, the pulmonary recruitment maneuver (PRM) can also facilitate the removal of CO_2_ from the abdominal cavity by increasing positive airway pressure and intrathoracic pressure. PRM is more commonly used in clinical practice because it does not require drugs, specialized apparatus, or additional medical costs, unlike the other methods [16–17]. Several trials have described the advantages of PRM in patients undergoing laparoscopic operations compared to passive abdominal compression [18–20]. However, Kaloo et al. [9] reported no benefits of the PRM on postoperative patients suffering from PLSP. Thus, it remains unclear whether PRM is better than passive abdominal compression. Therefore, we systematically searched and analyzed the available studies to assess the efficacy and advantages of PRM over traditional abdominal compression in laparoscopic operations.

## Methods

This systematic review and meta-analysis complied with the PRISMA statement [21]. This systematic review was registered on Prospero with the registration number CRD42022315025.

### Eligibility criteria

This meta-analysis included randomized controlled trials (RCTs) irrespective of the language, year of publication, or sample size. Patients who had undergone any type of laparoscopic procedure were enrolled. In the control group, patients were subjected to abdominal compression to eliminate as much residual CO_2_ as possible, whereas, in the intervention groups, patients subjected to PRM alone with varying maximum inflation pressures or in combination with other interventions were included.

### Search strategy and data extraction

A systematic literature research of electronic databases, including PubMed, Embase, Web of Science, and Cochrane Central Register of Controlled Trials (CENTRAL), was conducted from the date of inception to January 31, 2022. References were imported into EndNote™ X9 software (Clarivate™, London, UK) for deduplication.The following search terms were used for PubMed: (“laparoscopy” [MeSH Terms] OR“laparoscopy”[All Fields]) AND (“shoulder pain”[MeSH Terms] OR (“shoulder”[All Fields]) AND ((“lung”[MeSH Terms] OR “lung”[All Fields] OR “pulmonary”[All Fields]) AND (“recruit”[All Fields] OR “recruitment”[All Fields] OR “recruitments”[All Fields]) AND (“maneuver”[All Fields] OR “maneuvered”[All Fields] OR “maneuvering”[All Fields] OR “maneuverings” [All Fields] OR “maneuvers”[All Fields]).

The titles and abstracts of the articles were screened, and the full texts of relevant articles were studied further. DX and LH independently reviewed all resulting search entries against the inclusion and exclusion criteria and then extracted data from the included studies using a data extraction form. Data on the author’s name, year of publication, type of surgery, interventions used and relevant outcomes were collected from each study.

### Assessment of the risk of bias

The online bias-assessment tool RoB-2 was used to assess the quality of included studies [22]. This tool evaluated the risk of bias in each included study based on the following aspects: (1) randomization process; (2) deviations from intended interventions; (3) missing outcome data; (4) measurement of the outcome; (5) selection; (6) selective reporting (reporting bias) and (7) other bias. The risk of bias in each item was categorized as low, high, and some concern.

### Statistical analysis

Statistical meta-analysis was performed using the statistical software Rev Man version 5.4 (The Cochrane Collaboration, Copenhagen, Denmark). Confidence intervals were set at 95%. The mean difference (MD) and 95%CI were the principal summary measures for pooled continuous and normally distributed outcomes. Zero-to-hundred pain scale scores for pain were converted to zero-to-ten scale scores to facilitate statistical analysis. The odds ratios (OR) and 95%CI were the principal summary measures for pooled dichotomous data. Summary measures were considered statistically significant if the 95% CI for the mean difference excluded zero and if the 95% CI or the odds ratios excluded 1.

The I^2^ statistic was used to quantify heterogeneity in the pooled results. Significant heterogeneity was defined as an I^2^ value of > 50%. The Der Simonian–Laird random-effects model was used if significant heterogeneity was detected in the methodologies of the included studies. The median and interquartile range (IQR) were transformed to mean and standard difference (SD) [23, 24].

## Results

**Fig. 1:**
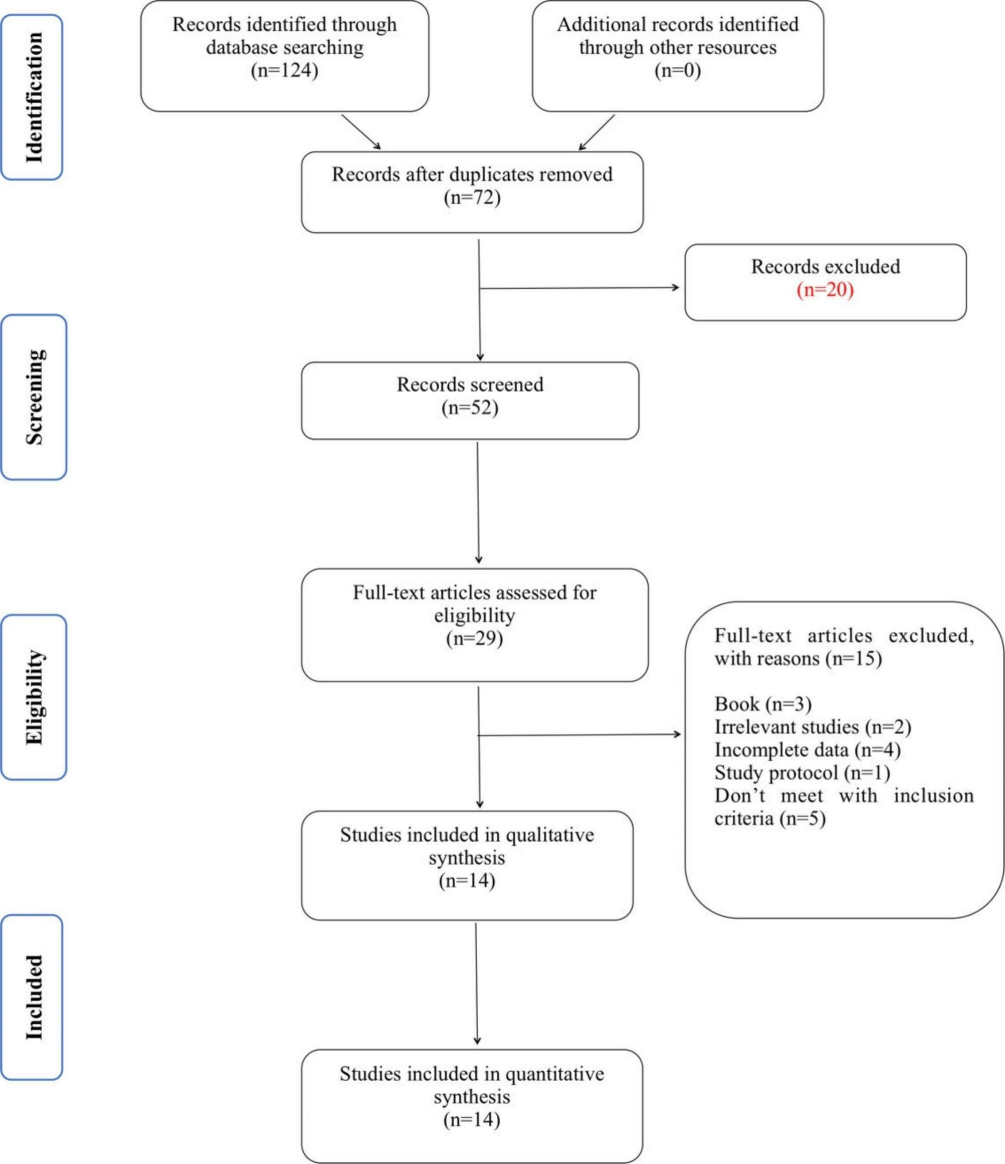
PRISMA flow diagram

We searched the databases PubMed, EMBASE, Web of Science and Cochrane Central Register of Controlled Trials (CENTRAL) to obtain a total of 124 results. The full texts of 29 articles were examined in detail. Two researchers (DX and LH) reviewed all the full texts. Finally, we included 14 RCTs with a total of 1504 participants were included in the meta-analysis (Fig. 1).

### Characteristics of the included studies

The details of included studies are presented in Table 1. Eleven studies compared the control group (passive abdominal compression) and PRM alone [16–17, 25–36]. Three studies compared passive abdominal compression in combination with intraperitoneal saline [33, 35−36].

**Table 1:** Characteristic of included studies, PRM, pulmonary recruitment maneuver; SI, saline instillation; LC, laparoscopic cholecystectomy; N/A, not applicable

Author; date	Group	Patient number	Type of operation	CO_2_ pressure (mmHg)	PRM Treatment
Kihlstedt PE.; 2021 [16]	Control PRM (40cmH_2_O)	71 76	LC	12	During one-minute of pressure-controlled ventilation, the patient received 6 breaths with a total pressure of 40 cm H_2_O in supine position
Ryu KH.; 2019 [29]	Control SI SI + PRM (40cmH_2_O)	48 48 48	gynecologic surgery	14	5 manual pulmonary hyperinflations using positive inspiratory pressure, each inflation was maintained at an end-inspiratory plateau pressure of 40 cmH_2_O for 5 s
Lee J.; 2020 [28]	Control PRM (30cmH_2_O)	42 42	gynecologic surgery	10	5 manual pulmonary inflations for 5 s with pressure of 30 cm H_2_O
Kiyak H.; 2019 [34]	Control PRM (40cmH_2_O) PRM + semi-fowler	41 33 32	gynecologic surgery	N/A	5 manual inflations at a maximum pressure of 40cmH_2_O in the neutral position or semi-fowler position
Davari-Tanha F.; 2019 [25]	Control PRM (60cmH_2_O)	70 70	gynecologic surgery	N/A	5 manual pulmonary inflations at a maximum pressure of 60 cm H_2_O
van Dijk JEW.; 2018 [33]	Control SI + PRM (40cmH_2_O)	88 89	gynecologic surgery	14	5 pulmonary insufflations with a pressure 40 cm H_2_O
Güngördük K.; 2018 [26]	Control PRM (40cmH_2_O)	52 54	gynecologic surgery	20	2 manual inflations to a maximum pressure of 40cmH_2_O, each positive inflation was held for 5s
Ryu K.; 2017 [35]	Control SI + PRM (40cmH_2_O) SI + PRM (60cmH_2_O)	30 30 29	gynecologic surgery	14	5 manual pulmonary inflations at a maximum pressure of either 40 cmH_2_O (40cmH_2_O group) or 60 cmH_2_O (60 cmH_2_O group).
Tsai HW.; 2013 [36]	Control SI + PRM (60cmH_2_O)	50 50	gynecologic surgery	15	After normal saline instillation, 5 manual pulmonary inflations at a maximum pressure of 60 cm H_2_O
Khanna A.; 2013 [27]	Control PRM (60cmH_2_O)	39 37	LC、hernia	14	2 manual inflations to a maximum pressure of 60cmH_2_O, each positive pressure inflation for 5 s
Tsai HW.; 2011 [31]	Control PRM (60cmH_2_O) INSI	51 53 54	gynecologic surgery	15	a pulmonary recruitment maneuver consisting of 5 manual pulmonary inflations was performed with a maximal pressure of 60cmH_2_O.The fifth positive-pressure inflation for 5 s.
Sharami SH.; 2010 [30]	Control PRM (40cmH_2_O)	64 67	gynecologic surgery	15	5 manual pulmonary inflation at a positive pressure 40cmH_2_O, fifth was held for 5s.
Tsai HW.; 2010 [32]	Control PRM (60cmH_2_O) SI	30 40 40	gynecologic surgery	N/A	A pulmonary recruitment maneuver consisting of 5 manual pulmonary inflations was performed with a maximum pressure of 60 cm H_2_O.
Phelps P.; 2008 [17]	Control PRM (60cmH_2_O)	46 54	gynecologic surgery	15	a PRM consisting of 5 manual pulmonary inflations was performed with a maximum pressure of 60 cm H_2_O, the fifth positive pressure inflation for approximately 5s

### Risk of bias in the included studies

**Fig. 2:**
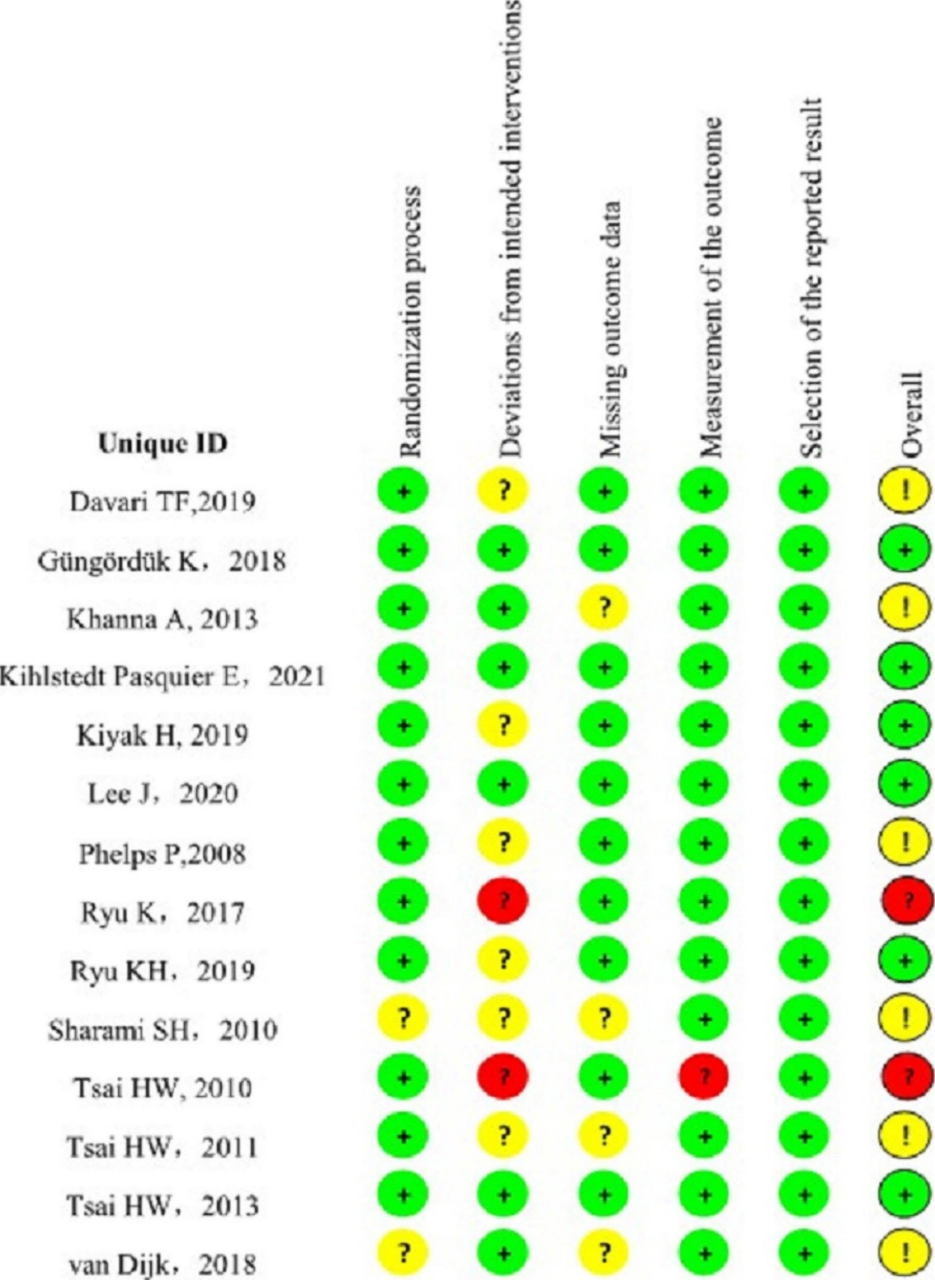
Risk of bias summary of included the trails: evaluation of bias risk items for each included study. Green circle, low risk of bias; red circle, high risk of bias; yellow circle, unclear risk of bias

Two authors (DX and LH) independently assessed the quality of the included studies using the online bias-assessment tool RoB-2 [22]. The risk of bias was classified as low, high, and some concern. Disagreements in risk assessment between the two authors were assessed and adjudicated by another independent reviewer (WYT). Figure 2 presents the risks of bias of the included references.

### The intensity of shoulder pain

Compared with the control group, PRM can significantly decrease the visual analog scales (VAS) scores of shoulder pain at 12 h (MD (95%CI) − 1.12 (–1.57, − 0.66), n = 801, *P* < 0.001, I^2^ = 88%), at 24 h (MD (95%CI) − 1.45(–1.74, − 1.16), n = 1180, *P* < 0.001, I^2^ = 78%), and at 48 h (MD (95%CI) − 0.97(–1.57, − 0.36), n = 780, *P* < 0.001, I^2^ = 85%).

**Fig. 3:**
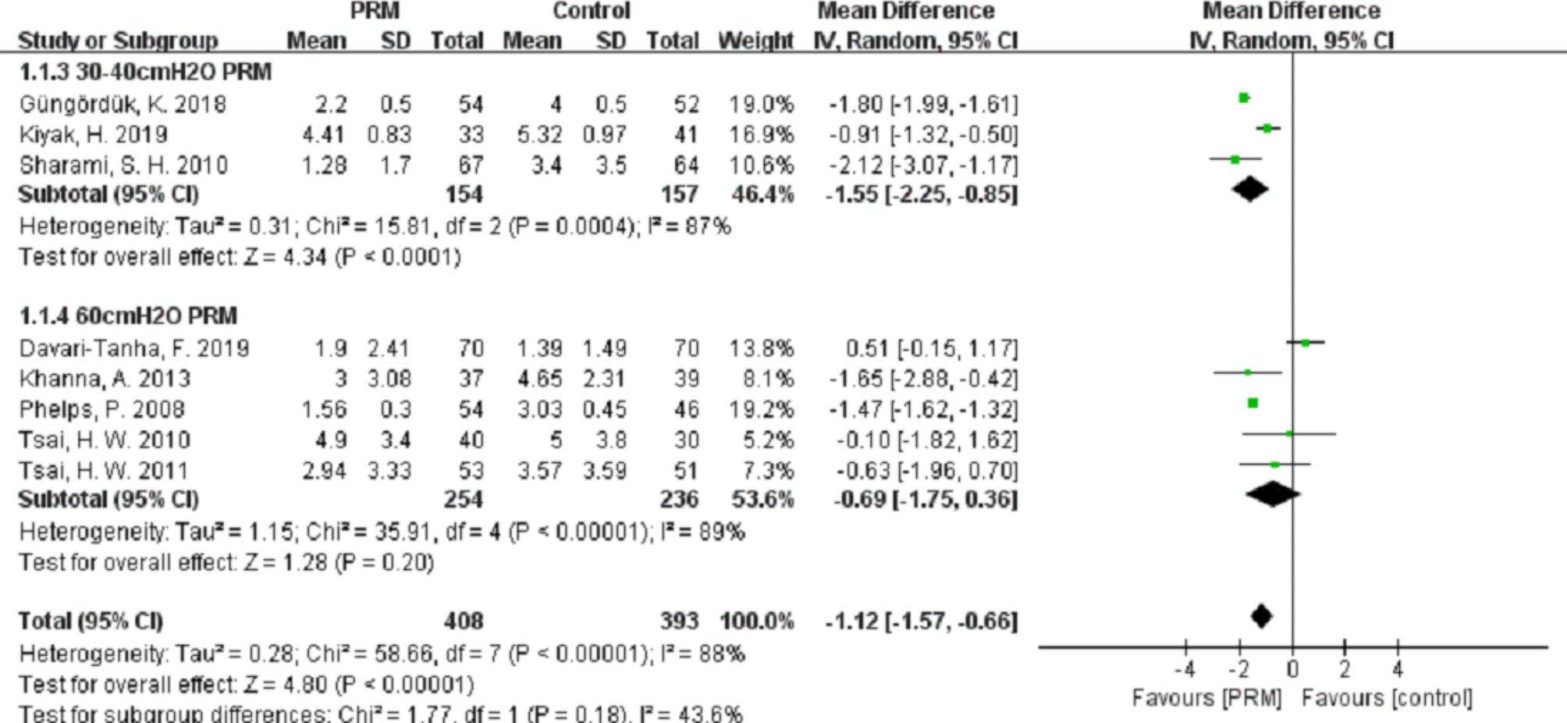
Forest plot of PLSP scores at 12 h after operation

**Fig. 4:**
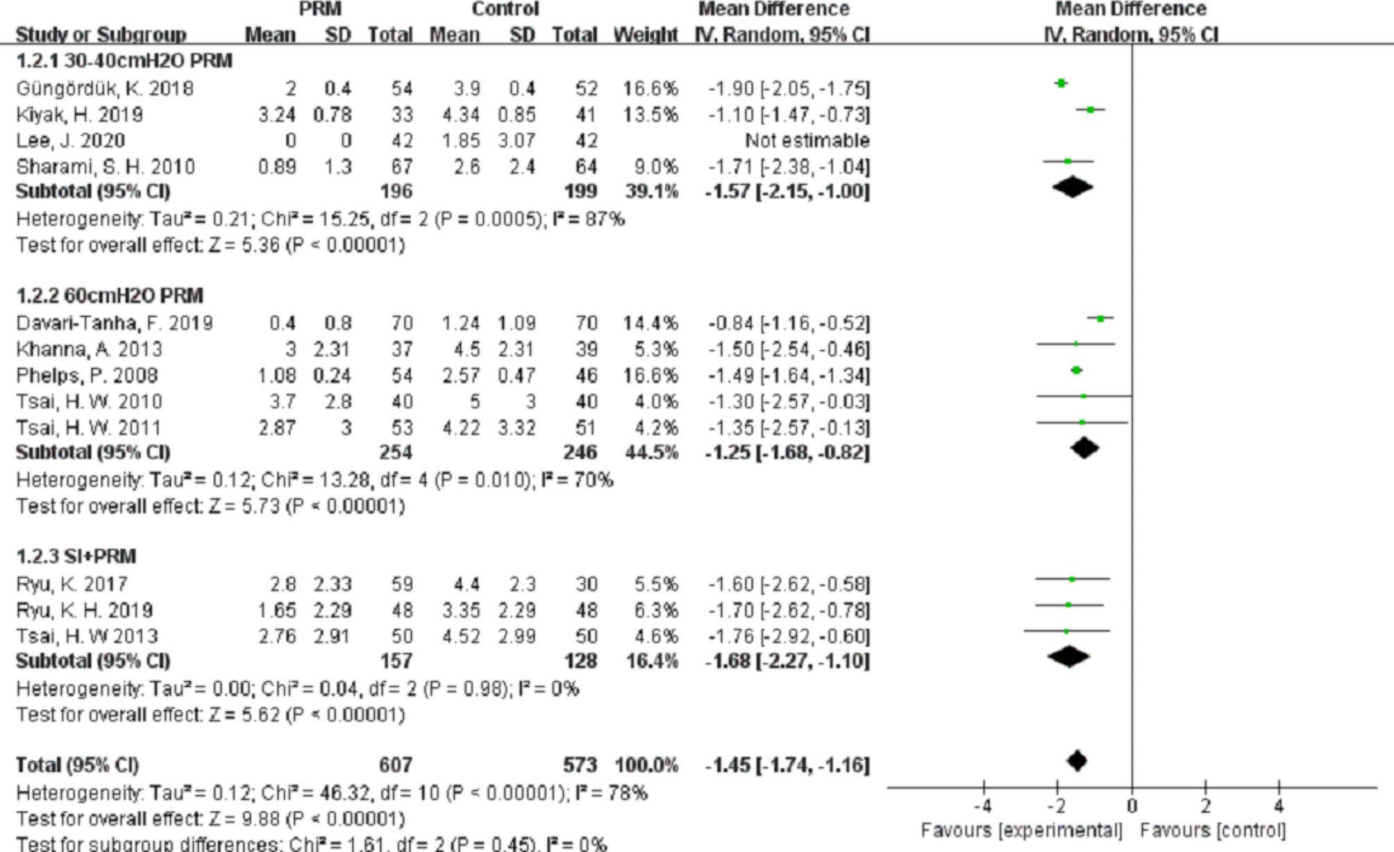
Forest plot of PLSP scores at 24 h after operation

**Fig. 5:**
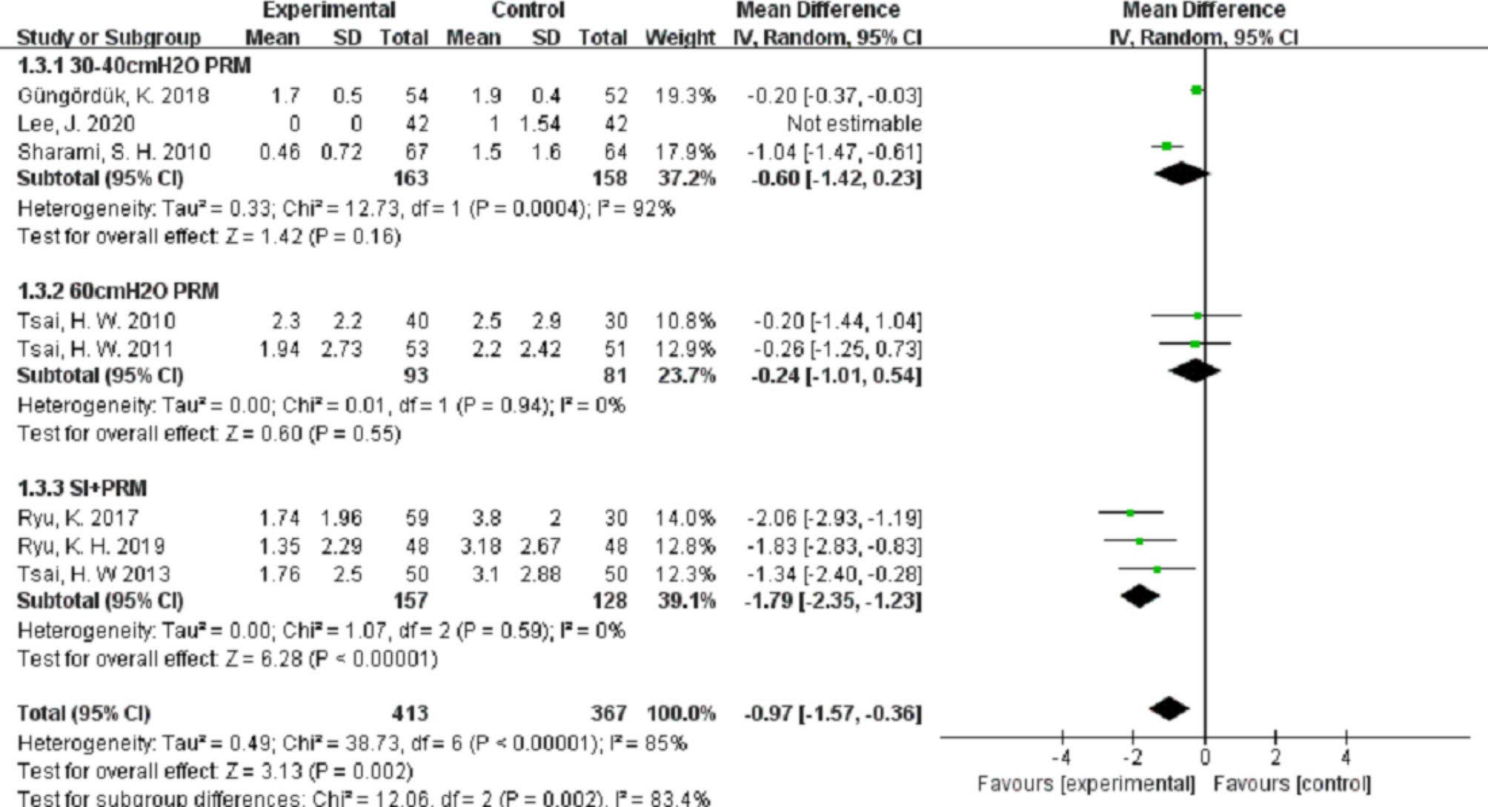
Forest plot of PLSP scores at 48 h after operation

However, we noted a considerable heterogeneity among the studies at different follow-up times (I^2^ = 88%, 78%, and 85% at 12 h, 24 h, and 48 h, respectively). This high heterogeneity could not be eliminated when we performed sub-group analyses using different pressures of PRM or in combination with IPSI (Figs. 3, 4 and 5), which indicated that the high heterogeneity was not related to our subgroup analysis.

### Sensitivity analysis

To further explore the possible cause of the high heterogeneity, we conducted a sensitivity analysis to assess the robustness of the synthesized results of repeat analyses by excluding one study at a time. We failed to find a difference in outcomes using this method. At 12 h after operation, the MD (95% CI) varied from − 1.42(–1.76, − 1.09) after excluding the study by Davari-Tanha et al. [25] to − 0.94(–1.58, − 0.31) after excluding the study by Güngördük et al.[26] At 24 h after operation, the MD (95% CI) varied from − 1.56 (–1.81, − 1.31) after excluding the study by Davari-Tanha et al. [25] to − 1.33 (–1.58, − 1.08) after excluding Güngördük et al. [26] At 48 h after operation, the MD (95% CI) varied from − 1.16 (–1.71, − 0.62) after excluding Güngördük et al. [26] to − 0.78 (–1.35, − 0.21) after excluding the study by Ryu et al. [35] (Tables 2, 3 and 4).

**Table 2:** The sensitivity of shoulder pain score at 12 h after operation

Removed study	MD	95% CI	*Z* value	*P* value	I^2^
Güngördük K. [26]	–0.94	(–1.58, − 0.31)	2.93	0.003	86%
Kiyak H. [34]	–1.15	(–1.66, − 0.65)	4.48	< 0.001	88%
Sharami SH. [30]	–0.99	(–1.48, − 0.51)	4.02	< 0.001	89%
Davari-Tanha F. [25]	–1.42	(–1.76, − 1.09)	8.33	< 0.001	74%
Khanna A. [27]	–1.07	(–1.55, − 0.58)	4.34	< 0.001	90%
Phelps P. [17]	–1.00	(–1.75, − 0.25)	2.62	< 0.001	90%
Tsai HW. [32]	–1.17	(–1.63, − 0.71)	4.95	< 0.001	89%
Tsai HW. [31]	–1.15	(–1.63, − 0.68)	4.77	< 0.001	89%

**Table 3:** The sensitivity analysis of shoulder pain score at 24 h after operation

Removed sutdy	MD	95% CI	*Z* value	*P* value	I^2^
Güngördük K. [26]	–1.33	(–1.58, − 1.08)	10.36	< 0.001	48%
Kiyak H. [34]	–1.51	(–1.81, − 1.20)	9.66	< 0.001	77%
Lee J. [28]	–1.45	(–1.74, − 1.12)	9.88	< 0.001	78%
Sharami SH. [30]	–1.43	(–1.73, − 0.51)	9.06	< 0.001	80%
Davari-Tanha F. [25]	–1.56	(–1.81, − 1.31)	12.36	< 0.001	62%
Khanna A. [27]	–1.45	(–1.75, − 1.15)	9.46	< 0.001	81%
Phelps P. [17]	–1.45	(–1.85, − 1.06)	7.25	< 0.001	80%
Tsai HW. [32]	–1.46	(–1.75, − 1.16)	9.62	< 0.001	80%
Tsai HW. [31]	–1.46	(–1.75, − 1.16)	9.58	< 0.001	81%
Ryu K. [35]	–1.44	(–1.74, − 1.14)	9.41	< 0.001	81%
Ryu KH. [29]	–1.43	(–1.74, − 1,13)	9.31	< 0.001	81%
Tsai HW. [36]	–1.44	(–1.73, − 1.14)	9.43	< 0.001	81%

**Table 4:** The sensitivity analysis of shoulder pain score at 48 h after operation

Removed sutdy	MD	95% CI	*Z* value	*P* value	I^2^
Güngördük, K. [26]	–1.16	(–1.71, − 0.62)	4.17	< 0.001	57%
Lee J. [28]	–0.97	(-1.57, − 0.36)	3.13	0.002	85%
Sharami SH. [30]	–0.96	(–1.73, − 0.19)	2.45	< 0.01	83%
Tsai HW. [32]	–1.06	(–1.73, − 0.40)	3.14	0.002	87%
Tsai HW. [31]	–1.08	(–1.76, − 0.40)	3.09	0.002	87%
Ryu K. [35]	–0.78	(–1.35, − 0.21)	2.68	0.007	80%
Ryu KH. [29]	–0.84	(–1.45, − 0.22)	2.66	0.008	84%
Tsai HW. [36]	–0.92	(–1.57, − 0.26)	2.75	0.006	86%

### Other outcomes

**Fig. 6:**
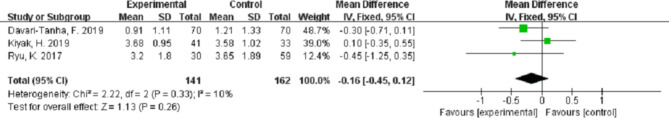
Forest plot of wound pain scores at 24 h after operation

**Fig. 7:**
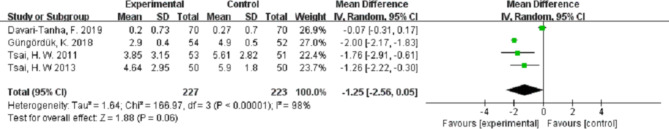
Forest plot of upper abdominal pain scores at 24 h after operation

**Fig. 8:**
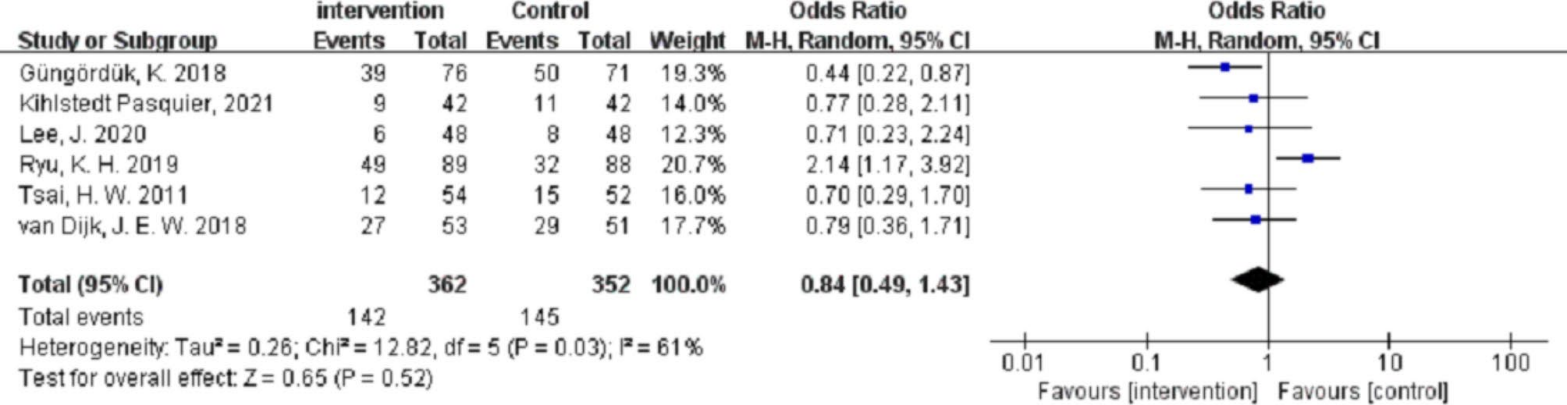
Forest plot of incidence of PONV

PRM did not reduce the intensity of wound pain [MD (95% CI) − 0.16 (–0.45 to 0.12), n = 303, *P* = 0.26, I^2^ = 10%] or upper abdominal pain [MD (95% CI) -1.25 (–2.56 to 0.05), n = 450, *P* = 0.52, I^2^ = 98%] at 24 h postoperatively and the incidence of postoperative nausea and vomiting(PONV) [OR (95% CI) 0.84 (0.49–1.43), n = 714, *P* = 0.52, I^2^ = 61%] (Figs. 6, 7 and 8).

## Discussion

Fourteen RCTs were included in our systematic review and meta-analysis comparing passive abdominal compression with PRM alone or in combination with IPSI. The results indicated that the application of PRM alone or in combination with IPSI could significantly decrease PLSP VAS scores at 12 h, 24 and 48 h postoperatively, compared with passive abdominal compression. However, this strategy was ineffective at reducing the intensity of postoperative wound pain, upper abdominal pain, and the incidence of PONV.

Although the mechanism of PLSP is not fully understood yet, it may involve the following hypotheses. First, carbonic acid that is converted from (CO_2_) by carbonic anhydrase on the surface of the diaphragm [16] can stimulate the phrenic nerve ending and transmits pain signals to the central nervous system (CNS) [37]. Moreover, the loss of suction from the liver and traction of the visceral ligament caused by residual gas in the enterocoeles can also directly cause pain [38]. It is suggested that residual CO_2_ in the abdominal cavity can remain for several days after laparoscopy [39–40] and postoperative shoulder pain may be correlated with the volume of CO_2_ under the right hemidiaphragm [12, 49]. The last hypothesis involves tissue trauma caused by the rapid insufflation of the pneumoperitoneum and the hyperdistention of the abdominal cavity, which results in overstretching of the diaphragmatic muscle fibers, traumatic straining of nerves, tearing of blood capillaries, and release of inflammatory mediators, which in turn elicits the referred pain to the shoulder [12, 41].

At the end of the surgery, PRM is often performed with manual positive-pressure ventilations, which not only inflate the lungs but also lower the diaphragm and increase intraperitoneal pressure. CO_2_ gas accumulated in the peritoneal cavity can be removed by increased intraperitoneal pressure, resulting in reduced irritation of the phrenic nerve or peritoneum and consequent shoulder pain. As indicated in our study, PRM could be easily performed and was an effective method for the prevention of PLSP. However, our study failed to show the benefit of PRM on the incision site and epigastric pain, as well as PONV. Pain at the wound and upper abdomen are mainly caused by surgical traumas such as skin incision and tissue excision, which are usually prevented and treated using oral analgesics, local infiltration, nerve block, and analgesic pump, and cannot be alleviated by reducing the residual CO_2_ gas in the cavity. As the incidence of PONV varies with several factors, including sex, history of PONV, smoking history, motion sickness, type of anesthetic and depth of anesthesia [55–57], the elimination of CO_2_ did not reduce the incidence of PONV.

It is worth noting that some other measures, including oral analgesics [42], intraperitoneal saline instillation (IPSI) [16], drain insertion [43], sodium bicarbonate sub-diaphragm irrigation [44], intraperitoneal anesthetic agents, and nerve-blocking agents [45–48] can also prevent PLSP. However, these methods not only require drugs and equipment but also involve additional medical costs. Moreover, they may even produce adverse effects. In contrast, the implementation of PRM is more convenient and simpler, which makes it worth popularizing. However, it should be noted that complications related to PRM, including barotrauma and hemodynamic deterioration, may occur when higher pressures are used [50–53]. Yilmaz et al. [54] suggested that a lower maximal inspiratory pressure of 15 cm H_2_O might be preferred to avoid the potential complications of PRM using higher pressures. Because of relatively fewer studies on the use of PRM at low pressures, we suggest that the optimal positive pressure of PRM, which minimizes the severity of PLSP and the incidence of adverse events, should be further explored further.

Compared with a previous study by Pergialiotis et al. [19], we included more types of laparoscopic surgeries besides gynecologic operations, such as cholecystectomy and hernia surgery. Moreover, our study analyzed more outcomes such as wound pain and the incidence of PONV. Therefore, our study provides more information and stronger evidence supporting the effect of PRM on PLSP.

This meta-analysis also have some limitations. First, despite the expansion of operation types, the final analysis only included two studies that were conducted on nongynecologic surgery patients. Further studies regarding to PLSP should investigate other types of laparoscopic operations in more detail. Second, there were high variations in medication for perioperative prophylactic analgesia in the included studies, which may affect the study results. Third, high heterogeneity was observed in our study, which may have resulted from different methodologies and clinical factors in the included studies, although we acknowledged this limitation and downgraded the quality of the evidence accordingly.

## Conclusion

Our study suggested that PRM is a feasible preventive measure for reducing the intensity of PLSP. However, the results of this meta-analysis should be interpreted with caution owing to the high heterogeneity between the analyzed studies. Moreover, the usefulness of PRM in other types of laparoscopic operations besides gynecological operations should be further explored further.

## Data Availability

materials. The datasets generated and analyzed during the current study are available from the corresponding author on reasonable request.

## Abbreviations

CI: Confidence interval
CNS: Central nerve system
CO2: Carbon dioxide
IPSI: Intraperitonial saline instillation
IQR: Inter quartile range
LC: Laparoscopic cholecystectomy
MD: Mean difference
N/A: Not applicable
OR: Odds ratio
PLSP: Post-laparoscopic shoulder pain
PONV: Postoperative nausea and vomiting
PRM: Pulmonary recruitment maneuver
SD: Standard difference
SI: Saline instillation
VAS: Visual analogue score
